# Comparative Study of Iron-Tailings-Based Cementitious Mortars with Incorporated Graphite Ore and Graphite Tailings: Strength Properties and Microstructure

**DOI:** 10.3390/ma16103743

**Published:** 2023-05-15

**Authors:** Jiale Zhang, Qi Wei, Na Zhang, Shuai Zhang, Yihe Zhang

**Affiliations:** 1 Engineering Research Center of Ministry of Education for Geological Carbon Storage and Low Carbon Utilization of Resources, China University of Geosciences (Beijing), Beijing 100083, China; 2 Beijing Key Laboratory of Materials Utilization of Nonmetallic Minerals and Solid Wastes, China University of Geosciences (Beijing), Beijing 100083, China; 3 National Laboratory of Mineral Materials, China University of Geosciences (Beijing), Beijing 100083, China; 4 School of Materials Science and Technology, China University of Geosciences (Beijing), Beijing 100083, China

**Keywords:** graphite tailings, graphite ore, mortar, strength properties, microstructure

## Abstract

Graphite ore and graphite tailings were blended into iron-tailings-based cementitious mortars, and their mechanical properties and microstructure were experimentally investigated. The flexural and compressive strengths of the resulting material were tested to compare the effects of graphite ore and graphite tailings as supplementary cementitious materials and fine aggregates on the mechanical properties of iron-tailings-based cementitious mortars. Additionally, their microstructure and hydration products were mainly analyzed using scanning electronic microscope and X-ray powder diffraction techniques. The experimental results showed that the mechanical properties of the mortar material incorporating graphite ore were reduced due to the lubricating properties of graphite ore. As a result, the unhydrated particles and aggregates were not tightly bound to the gel phase, making the direct application of graphite ore in construction materials unfeasible. In the iron-tailings-based cementitious mortars prepared in this work, the optimal incorporation rate of graphite ore as a supplementary cementitious material was 4 wt%. The compressive strength of the optimal mortar test block after 28 days of hydration was 23.21 MPa, and the flexural strength was 7.76 MPa. The mechanical properties of the mortar block were found to be optimal with a graphite-tailings content of 40 wt% and an iron-tailings content of 10 wt%, resulting in a 28-day compressive strength of 48.8 MPa and a flexural strength of 11.7 MPa. By observing the microstructure and XRD pattern of the 28-day hydrated mortar block, it was determined that the hydration products of the mortar with graphite tailings as an aggregate included ettringite, Ca(OH)_2_, and C-A-S-H gel.

## 1. Introduction

Graphite is a national strategic resource that finds wide applications in lubrication, wear resistance, corrosion resistance, electrical conductivity, and energy storage. It is an indispensable and crucial material in national defense, military, and mechanical industries. Simultaneously, as a nonrenewable resource, which plays a vital role in modern economic development. Consequently, the amount of graphite mined increases each year, leading to a considerable production of low-grade graphite ore and graphite tailings [1]. The flotation process is commonly used in the production of graphite, with each ton of graphite requiring 100 tons of water and 10 tons of raw graphite ore on average. Currently, China produces approximately 500,000 tons of crystalline graphite per year, generating over 6 million tons of graphite tailings after beneficiation. Unfortunately, efficient and large-scale applications of graphite-tailings processing capacities have not yet been developed. The traditional method of storing graphite tailings not only consumes significant land resources but also results in the release of dust and leakage from the tailings reservoir, leading to severe environmental pollution during adverse weather conditions [2,3]. Scholars have undertaken research to address the environmental pollution caused by tailings stockpiles, exploring comprehensive utilization methods, such as backfilling mines with tailings, planting and reclaiming tailings ponds, extracting valuable elements, and producing ecofriendly base materials for roads [4]. However, the economic benefits of these methods are limited due to the low utilization efficiency of graphite tailings. In recent years, experts have focused on developing building materials, largely by using graphite tailings, to manufacture materials such as no-burn bricks, cement, concrete, and ceramic products. These efforts aim to create economic benefits by leveraging the value of graphite tailings. The following lists the research of domestic and foreign scholars on the high-value-added utilization of graphite tailings:

Graphite postmining beneficiation is primarily divided into three main areas. High-quality and large-scale extracted graphite is utilized for preparing graphene, whereas medium-quality stone mineral ink is sorted for graphite. The remaining graphite tailings require urgent resource utilization. The main mineral composition of graphite ore is quartz, plagioclase, and graphite [5]. For instance, the lubricity and conductivity of graphite can be leveraged to prepare high-flow, conductive concrete and thermally conductive asphalt pavement materials. Additionally, expanded graphite can be utilized for preparing phase-change concrete [6,7].

Some researchers have mixed graphite tailings into concrete or mortar materials as admixtures and studied their mechanical properties. Wang et al. [8] used graphite tailings to prepare cement mortar and found that graphite tailings, with the optimal addition amount being 20 wt%, can significantly improve the freeze-resistance of cement mortar. In another study, Duan et al. [9] studied the feasibility of completely replacing a concrete sand aggregate with graphite tailings. Their results showed that the mechanical strength of the concrete samples underwent a parabolic change when there was an increase in the replacement rate, and the best replacement rate was 30%. It was also found that when the replacement rate exceeded 70%, the interfacial defects between the aggregate and matrix increased, and cracks were more likely to occur. Zhang et al. [10] used graphite tailings to replace sand and stone aggregates in reclaimed aggregate concrete. Their research showed that the optimal content of graphite tailings was 20 wt%, but when the content was between 10 wt% and 30 wt%, the water absorption rate, surface water content, mechanical properties, and elastic modulus of reclaimed aggregate concrete was significantly improved. Liu et al. [11] found that when steel-fiber-reinforced concrete was mixed with graphite tailings, the interfacial binding effect between the concrete aggregate and gel phase was enhanced; thus, the growth of microcracks was inhibited. They also found that the excessive addition of graphite tailings led to an uneven distribution of steel fibers, and ultimately reduced the mechanical properties of concrete. Because the main mineral composition of graphite tailings is quartz, muscovite, and calcite, the SiO_2_ content was very high. Therefore, Peng et al. [12] used graphite tailings as an alternative silicon source to prepare autoclaved, aerated concrete. Their research showed that the mechanical properties of aerated concrete were the best when the graphite tailings content was 60 wt% and the calcium–silicon ratio was 0.6.

Some scholars used graphite tailings for the synthesis and preparation of other high-value-added materials. Hu et al. [13] used graphite tailings as raw materials to prepare foamed ceramics using a high-temperature, self-foaming method. The residual carbon reacted with Fe_2_O_3_ and produced gas that was wrapped by the liquid phase to form a porous structure. Bai et al. [14] used graphite tailings as the initiator of Fenton reactions and found that the mesoporous structure of graphite tailings provided it with good adsorption properties, and the Fe_2_O_3_ wrapped in it was able to initiate the Fenton reaction. Fu et al. [15] used a co-pyrolysis method to combine graphite tailings with coking coal to produce a composite anode and found that the combination of the two realized the lamellar reconstruction of graphite tailings and improved the transfer efficiency and cycling stability of lithium ions in the anode.

Liu and Wang et al. [16,17] investigated the electrical conductivity and mechanical properties of asphalt concrete with added graphene and carbon fibers. The study revealed that the optimal content of conductive medium in the asphalt concrete was 0.2 wt% of carbon fiber and 1.5 wt% of graphene. The test results indicated that the addition of graphene reduced the resistivity of the concrete, and the graphene particles filled the gaps in the conductive paths, thereby extending them. Li et al. [18] conducted a study on the use of graphene to reinforce concrete and develop high-strength concrete. Their research revealed that the content of ettringite, a hydration product of the cementing material, increased with higher graphene contents. Additionally, the authors found that when the graphene content reached 0.03 wt%, the flexural strength of the composite material increased by 40%. Based on the above literature, it is known that graphite tailings and graphene have been utilized in both cement concrete and asphalt concrete.

With the rapid economic development and the continuous expansion of urban areas, urbanization in China has accelerated, leading to an increasing number of public and residential buildings and a subsequent growth in construction areas. According to the “China Building Energy Consumption Research Report 2022,” the total energy consumption attributed to buildings and construction in China amounted to 2.27 billion tons in 2020, accounting for 45.5% of the country’s total energy consumption. Specifically, the energy consumption related to the production of building materials and the construction phase reached 1.2 billion tons. Consequently, the use of lower-carbon materials to replace cement in concrete preparation became a hot topic in order to achieve China’s carbon peaking and carbon neutrality goals.

Ibrahim et al. [19] employed silica fume as a supplementary cementitious material to partially substitute cement in the preparation of date-palm-fiber (DPF)-reinforced concrete. The inclusion of DPF had an adverse impact on concrete strength and porosity. However, the study revealed that by adding 2 wt% of DPF and replacing 10 wt% of cement with silica fume, the resulting concrete exhibited superior mechanical properties and a well-developed microstructure. Golewski [20] investigated the combination of nano-silica (NS) and fly ash (CFA) as a cement substitute to enhance concrete performance. The research demonstrated that the synergistic utilization of NS and CFA led to notable improvements in mechanical parameters and the concrete microstructure. Remarkably, the replacement ratio of cement with the NS and CFA mixture reached as high as 30 wt%. Furthermore, Abadel et al. [21] studied two solid waste materials with potential cementitious properties: recycled building cementitious materials (RCCMs) and red mud (RM). The researchers processed the RCCMs at high temperatures to obtain dehydrated cement powder (DCP). The study confirmed that the optimal dosage of DCP and RM was 7.5 wt% each. Notably, by replacing traditional cement with these additives, the 28-day compressive strength of concrete test blocks increased by 42.2%.

The above researchers used solid waste materials instead of cement for the synthesis and preparation of concrete materials, which not only improved the mechanical properties and microstructures of the materials, but also reduced the carbon footprint of cement-based materials, providing a feasible solution for the production of ecofriendly concrete. This also provided a new idea for the rational use of graphite ore and graphite tailings in this paper. Herein, the purpose of this paper is to present a practical idea for the resource utilization of graphite tailings. Iron-tailings-based cementitious mortars were prepared by combining graphite ore and graphite tailings with traditional solid-waste materials such as fly ash, blast-furnace slag, and iron tailings. By comparing the particle size of graphite ore and graphite tailings and assessing the mechanical properties of cementitious mortars and concrete prepared with graphite ore and graphite tailings, the feasibility of using graphite ore and graphite tailings as supplementary cementitious materials and fine aggregates was explored. Additionally, an analysis was conducted to determine the factors contributing to the changes in hydration products and mechanical properties of the cementitious mortars that incorporated graphite ore and graphite tailings.

## 2. Experimental Section

### 2.1. Raw Materials

**Figure 1:**
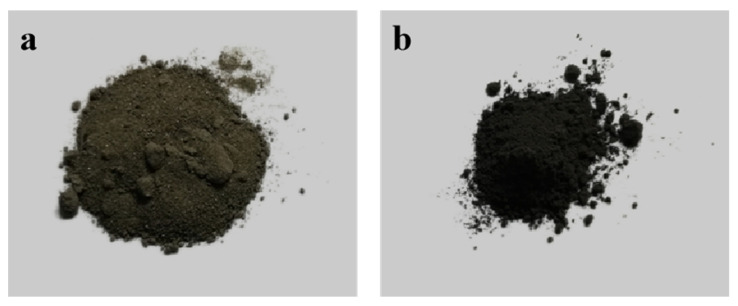
Appearance of graphite tailings (**a**) and graphite ore (**b**).

**Figure 2:**
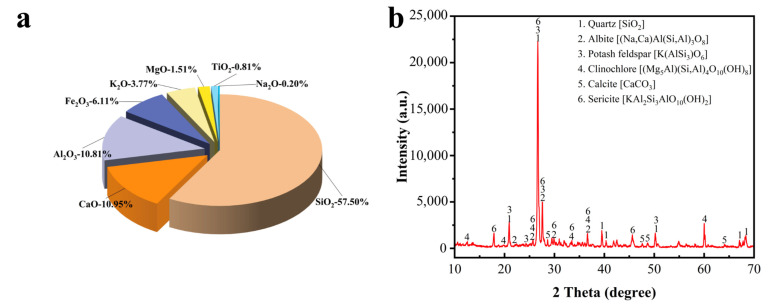
XRF analysis result (**a**) and XRD pattern (**b**) of graphite tailings.

**Figure 3:**
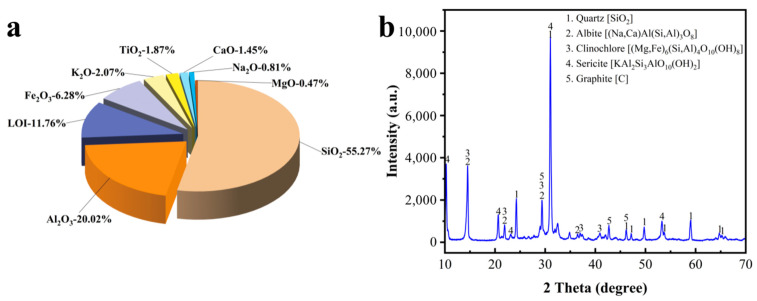
XRF analysis result (**a**) and XRD pattern (**b**) of graphite ore.

Based on the principle of solid waste to maximize utilization of resources, the raw materials selected for this experiment were graphite ore, graphite tailings, fly ash, blast-furnace slag powder (S95), ordinary Portland cement, iron tailings, and desulfurized gypsum. The sources of the materials are shown in Table 1. The appearance, chemical composition, and XRD patterns of graphite tailings and graphite ore are shown in Figure 1, Figure 2 and Figure 3. It can be seen from Figure 2 that the main chemical components of the graphite tailings used in the test include SiO_2_, Al_2_O_3_, Fe_2_O_3_, and CaO, among which the SiO_2_ content was as high as 57.5% and the loss on ignition was 4.33%. Through the analysis of the diffraction peaks in the XRD patterns, it was found that the SiO_2_ in graphite tailings mainly exist in the form of quartz minerals. In addition, the characteristic peaks of albite, sericite, potash feldspar, calcite, and clinochlorite were also found in the pattern. The XRD diffraction peak analysis results are basically consistent with the chemical composition obtained through XRF analysis. It can be seen from Figure 3 that the main chemical components of the graphite ore used in the test include SiO_2_, Al_2_O_3_, Fe_2_O_3_, and K_2_O, among which the SiO_2_ content was 55.27% and the loss on ignition was 11.76%. The ore contained a typical nonmetallic graphite composition with a C content of 7.05 wt%. The main mineral composition was graphite, quartz, albite, sericite, clinochlorite, etc., and the content of silicate minerals was high.

### 2.2. Preparation of Mortars

**Figure 4:**
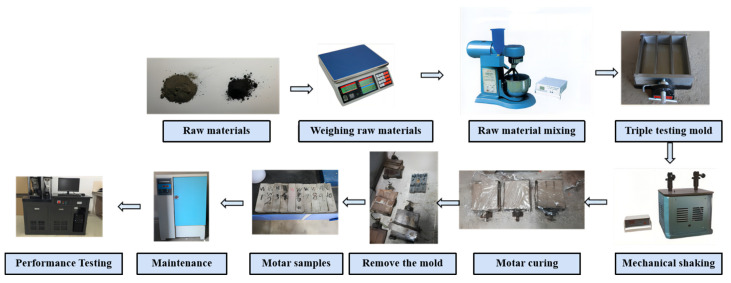
Cementitious mortar preparation process.

The experimental apparatus included a planetary mortar mixer (JJ-5), a vibrating table, and a 40 × 40 × 160 mm triple mold. The cementitious mortars were prepared at a water-to-binder ratio of 0.4, following the experimental design outlined in Table 2, Table 3 and Table 4. Table 2 is the specific ratio of the graphite-ore-incorporated mortar (GOM) as a supplementary cementitious material. Table 3 is the specific ratio of the graphite-tailings-incorporated mortar (GTM) as a supplementary cementitious material. Table 4 is the specific ratio of the graphite-tailings-replaced iron-tailings mortar (GTTM) as a fine aggregate. The specific preparation process is shown in Figure 4 [22].

(a) Mortar preparation.

The raw materials were poured into a mortar mixer at 300 rpm and the mixing time was 120 s.

(b) Mortar forming.

The mold was fixed on the vibration table, and the oil was brushed on the inner wall of the mold. After the mixing process was completed, the vibration table was opened, and the mixed cementitious mortar was poured into the mold and vibrated for 120 s. After the vibration stopped, the excess mortar was scraped off, and the preservative film was wrapped to prevent water loss.

(c) Mortar block curing.

After 24 h of curing, the mortar test block was placed in a constant-temperature and humidity curing box. The curing humidity was >90%, and the temperature was 22~23 °C.

(d) Mortar block test.

After the mortar blocks had been hydrated for 3 days, 7 days, and 28 days, they were taken out for flexural and compressive strength tests.

### 2.3. Testing Methods

The molding, curing, and testing of the mortar test block were carried out according to the GBT 17671-1999 standard [22], using a cube test block with a size of 40 mm × 40 mm × 160 mm. After the mortar block had cured for 3, 7, and 28 days, its flexural and compressive strengths were measured. The flexural strength was determined using the KZJ-500 cement electric bending test machine (manufactured by Shenyang Changcheng Electromechanical Equipment Factory, Shenyang, China), whereas the compressive strength was measured using an electrohydraulic servo universal testing machine. A three-point bending testing method was used to test the samples at a loading rate of 50 N/s.

The samples with curing ages of 3 days and 7 days were immersed in ethanol for 72 h to terminate the hydration process, and then dried for 12 h under a vacuum condition of 55 °C. After gold sputtering, the samples were photographed using an SU-8020 field emission scanning electron microscope (manufactured by Hitachi Ltd., Tokyo, Japan) to obtain SEM images.

The constituent analysis of the specimens was carried out via X-Ray powder diffraction (XRD) (D8, ADVANCE, manufactured by Bruker Corporation, Bremen, Germany) using Cu Kα radiation (λ = 1.54056 Å) and operating at 40 kV and 40 mA. The qualitative analysis examined the hydration products with a scanning rate of 6 degree/min, in a 2θ range of 5–70 degrees.

## 3. Results and Discussion

### 3.1. Feasibility of Using Graphite Ore and Graphite Tailings as Supplementary Cementitious Materials

**Figure 5:**
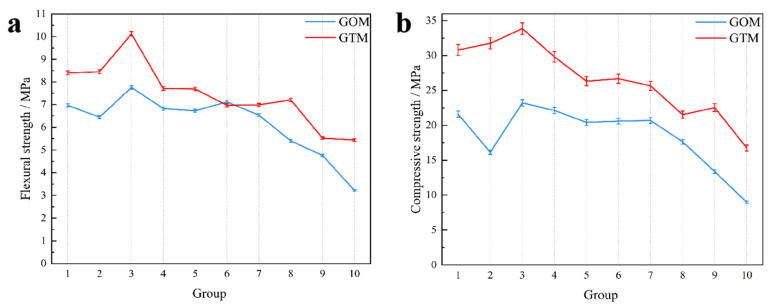
The 28-day flexural strength (**a**) and compressive strength (**b**) of mortars incorporating graphite ore and graphite tailings as supplementary cementitious materials.

Figure 5 depicts the changes in the mechanical properties of GOM and GTM after 28 days of curing. The flexural and compressive strengths of the mortar blocks obtained by mixing graphite ore with 52.5 Portland cement were lower than those of mortar blocks obtained by mixing graphite tailings with 42.5 Portland cement. With increases in the contents of graphite ore and graphite tailings, the flexural and compressive strengths of the mortar blocks decreased significantly. It was noticed that the strength of the GOM basically does not exceed that of GTM. This phenomenon could be attributed to the presence of the graphite phase within graphite ore, which imparted lubricity to the material. This lubricity, in turn, hindered the close bonding between the internal aggregate and the gel phase, leading to a significant reduction in the mechanical properties of the GOM.

**Figure 6:**
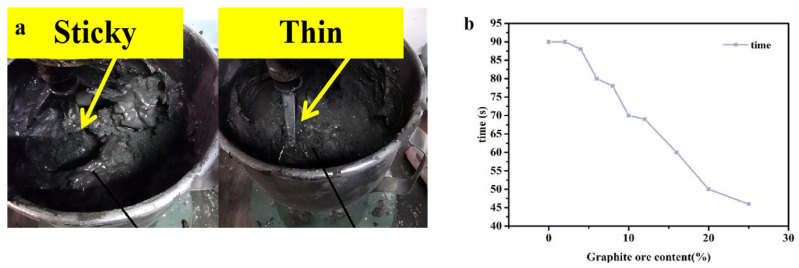
State and rheology of graphite-ore-incorporated mortar: (**a**) graphite-ore-incorporated mortar mixing state; (**b**) the time taken for graphite-ore-incorporated mortar to change from sticky to thin with different contents of graphite ore.

In order to verify the above speculation, based on the change trend of the mechanical properties of graphite-ore cementitious mortar, the influence of the lubricity of graphite ore on the rheological properties of mortar was further studied. The rheological properties of the cementitious mortar mixed with graphite ore are presented in Figure 6. After a few moments, its fluidity suddenly increased, causing the mortar to detach from the stirring paddle. Figure 6b illustrates that, as the graphite ore content increased, the time required for the mortar to transition from sticky to thin during the mixing process became shorter. This indicates that the lubrication property of graphite ore had a significant impact on the fluidity of the slurry. Therefore, it was concluded that the addition of graphite ore as a supplementary cementitious material should not exceed 12 wt% in the mortar due to its high lubricity, which could result in a substantial decrease in the mechanical strength of the mortar blocks.

**Figure 7:**
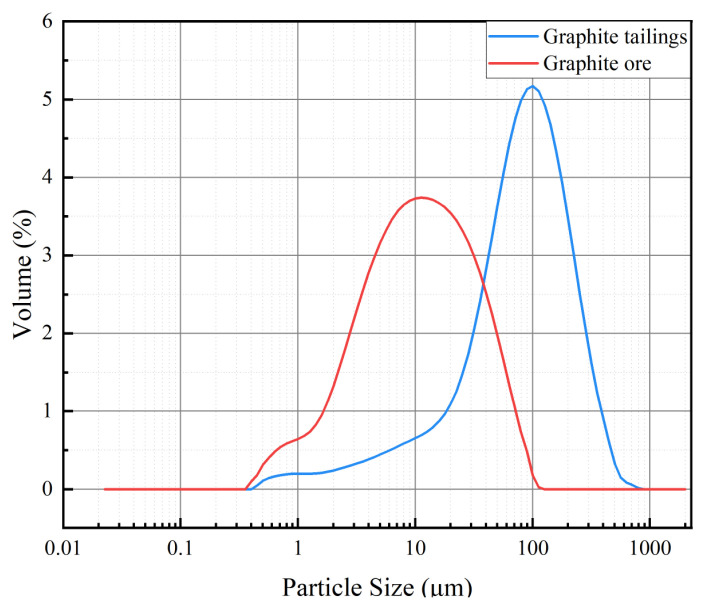
Particle size distributions of graphite tailings and graphite ore.

To investigate the potential of incorporating graphite tailings into mortar as a fine aggregate, the particle size distributions of both graphite ore and graphite tailings were measured. Figure 7 reveals that the particle size of graphite ore was mainly distributed at 10 μm, whereas that of graphite tailings were primarily in the range of 100 μm, which was ten times larger than the particle size of graphite ore and similar to the particle size of river sand [23]. This result indicated that the particle size of graphite ore was excessively fine and could only function as a supplementary cementitious material in mortar, whereas the graphite tailings held potential to replace river sand as a fine aggregate in mortar.

### 3.2. Feasibility of Using Graphite Tailings as a Fine Aggregate

The use of graphite tailings as a replacement for river sand in concrete depended on whether the physical properties of graphite tailings were similar to those of the river sand. Simonsen et al. [24] conducted a physical properties test on graphite tailings. It was found that the basic physical properties of graphite tailings were similar to those of river sand, as shown in Table 5. The bulk density and fineness modulus of graphite tailings were slightly smaller than those of river sand, suggesting that the average particle size of graphite tailings was smaller compared to river sand. This characteristic allowed graphite tailings to effectively fill the pores in the mortar block structure, potentially leading to better development of mechanical strength during hydration.

**Figure 8:**
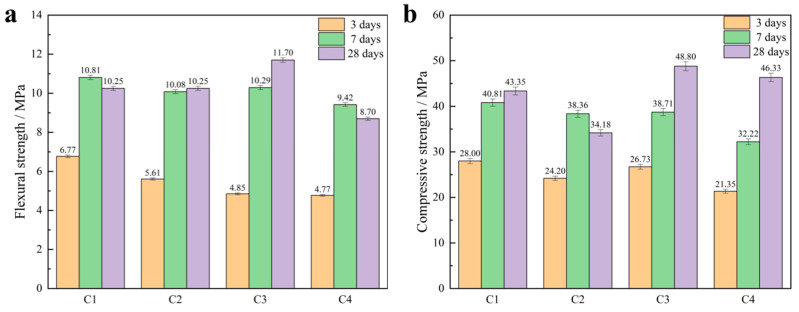
Mechanical properties test results: (**a**) flexural strengths of mortar with graphite tailings replacing iron tailings as fine aggregates; (**b**) compressive strengths of mortar with graphite tailings replacing iron tailings as fine aggregates.

Figure 8 depicts the flexural and compressive strengths of mortar samples with graphite tailings replacing iron tailings as fine aggregates (GTTM) after 28 days of curing. The results demonstrated that the mechanical properties of GTTM were excellent, particularly when the content of graphite tailings was 40 wt%. The 28-day compressive and flexural strengths of GTTM at this content (C3 group in Figure 8) reached 48.8 MPa and 11.7 MPa, respectively, which were significantly higher than those of other groups. It indicated that using graphite tailings as fine aggregates could result in much stronger mechanical properties for the cementitious mortar.

### 3.3. Microstructure Analysis of GOM and GTTM

**Figure 9:**
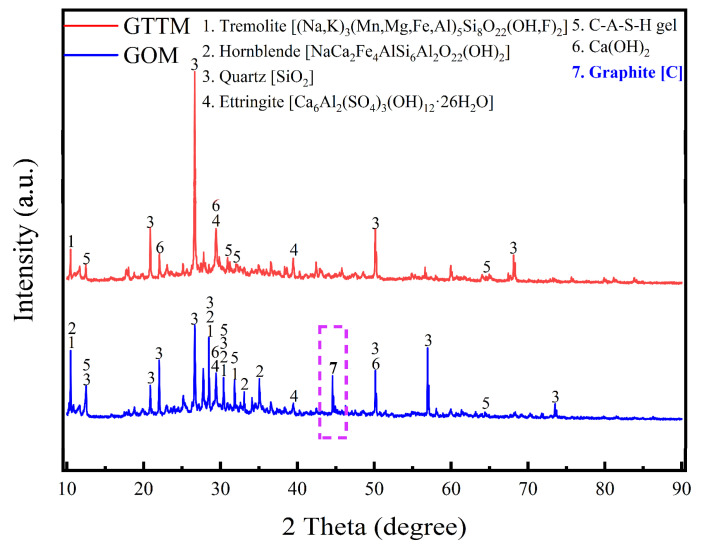
XRD patterns of cementitious mortar incorporating 4 wt% of graphite ore as supplementary cementitious material (GOM) and cementitious mortar incorporating 40 wt% of graphite tailings as fine aggregate (GTTM) after hydration for 28 days.

Figure 9 shows the XRD patterns of graphite-ore-incorporated mortar and GTTM after curing for 28 days. The wide peaks around 25–35° corresponded to amorphous C-A-S-H gels, which were the main hydration products of cementitious materials that incorporated iron tailings [25,26]. The peaks near 29° and 50° corresponded to the presence of the hydration product Ca(OH)_2_. In addition, characteristic peaks of ettringite, a mineral phase commonly presented in cement hydration systems, appear around 28° and 39°.

The presence of carbon was detected in the XRD pattern of GOM, which was consistent with the above mechanical and rheological properties results. Due to the presence of carbon, the lubricity of graphite ore led to the formation of microcracks and ultimately reduced the mechanical strength of the mortar block. This finding highlighted the importance of carefully selecting graphite materials and optimizing the graphite dosage in cementing systems.

**Figure 10:**
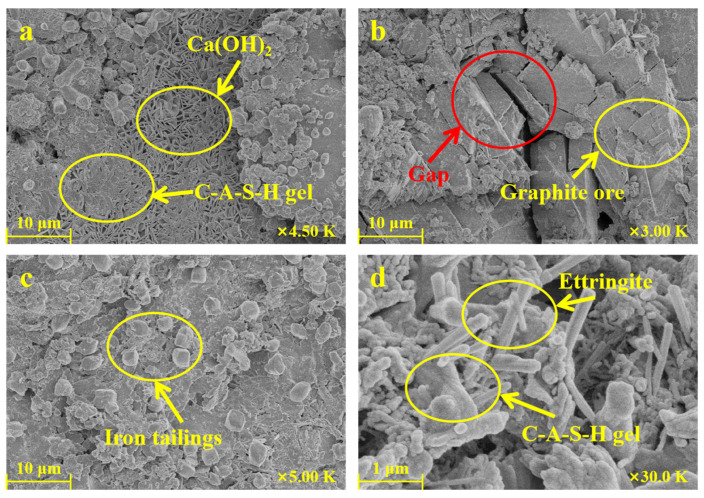
SEM images of cementitious mortar incorporating 4 wt% of graphite ore as supplementary cementitious material after hydration for 28 days. (**a**) the hydration products C-A-S-H gel and Ca(OH)_2_ in GOM; (**b**) unreacted graphite phase and generated microcracks in GOM; (**c**) iron tailings particles as fine aggregate in GOM; (**d**) the hydration product ettringite and C-A-S-H gel in GOM.

Figure 10 shows the SEM images of a GOM (A3 sample in Table 2) hydrated for 28 days. It can be seen in Figure 10a that the hydration products C-A-S-H gel and Ca(OH)_2_ were filling the pores. Additionally, Figure 10b shows the unreacted graphite ore. Due to the lubricity of the layered graphite in the graphite ore, the bonding force between the hydration product and the aggregate was weakened, leading to microcracks in the mortar block, which can negatively affect the strength growth of the material. This observation was in agreement with the results of the above XRD patterns, and this conclusion was also quoted in Section 3.1, where it was found that exceeding a graphite ore content of 12 wt% can lead to an excessively high amount of graphite in the cementitious mortar. This can result in an excessive number of microcracks inside the mortar block and lead to a significant reduction in mechanical strength. Figure 10c shows the iron tailings particles, which were seen as aggregates and are closely combined with the C-A-S-H gel. Figure 10d shows that the hydration products, columnar ettringite and amorphous C-A-S-H gel, tightly filled the pores between the aggregates, allowing the later strength of the material to be developed.

**Figure 11:**
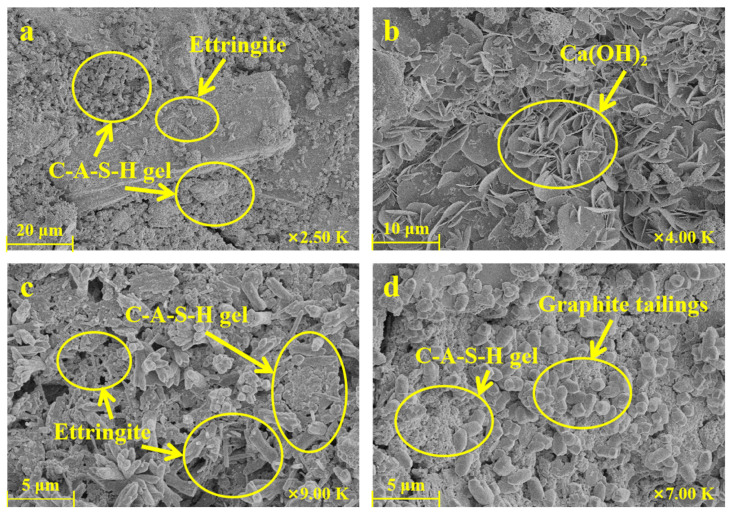
SEM images of cementitious mortar incorporating 40 wt% of graphite tailings as fine aggregate after hydration for 28 days. (**a**) the hydration products ettringite and C-A-S-H gels in GTTM; (**b**) the hydration products Ca(OH)_2_ in GTTM; (**c**) the C-A-S-H gels were distributed around the columnar ettringite; (**d**) the C-A-S-H gels were attached to the graphite tailings particles.

Figure 11 shows SEM images of GTTM (C3 sample in Table 4) hydrated for 28 days. Unlike the GOM sample, there were no obvious voids or microcracks around the large aggregate particles (Figure 11a). The main reason for its excellent mechanical properties was that the C-A-S-H gel, ettringite, and graphite tailings particles were closely connected, forming a dense matrix for the full development of the mortar strength in the later stage of hydration. Figure 11b displays a large number of calcium hydroxide flakes produced due to the cement hydration. Figure 11c,d shows that the C-A-S-H gels were distributed around the columnar ettringite and attached to the graphite tailings particles. No obvious microcracks are visible, which confirms the previous conclusion that graphite tailings are more suitable as fine aggregates to be incorporated in the cementitious mortar. The graphite tailings could fill holes in the mortar structure and enhanced the mortar’s mechanical properties.

The comparison between Figure 10 and Figure 11 clearly indicates that there was better densification between the graphite tailings and the C-A-S-H gels, which is consistent with the results from other studies [25,26,27]. When a graphite tailings content of 40 wt% was incorporated, hydration products, such as columnar ettringite and amorphous C-A-S-H gels, were present in large quantities, combining with the aggregate to densify the mortar structure, further increasing the mechanical strength of the cementitious mortar. This was close to the experimental conclusion of Zhang et al.’s research [10]. They found that the optimum content of graphite tailings was about 30 wt%. When the content of graphite tailings was more than 70 wt%, the interface defect of the aggregate matrix increased, so cracks inside the material were more likely to extend the interface. Additionally, the pore structure and internal fracture spaces were filled with new crystals, such as Ca(OH)_2_ and ettringite, which were further covered with a thick layer of amorphous C-A-S-H gels, resulting in a dense mortar structure. In contrast, due to the lubricity possessed by the graphite itself, the mortar material that was mixed with graphite ore led to cracks between the iron tailings aggregate and hydration products, which affected the subsequent mechanical strength development of the material.

## 4. Conclusions

Through mixing graphite ore and graphite tailings into the cementitious mortars and exploring the laws affecting the changes in the mechanical strength of the cementitious material, we obtained the following conclusions:

(1) The 28-day flexural and compressive strengths of GOM were considerably lower than those of GTM due to the strong lubricity of graphite ore. This lubricity can cause relative sliding between the aggregate and hydration products, resulting in cracks that can negatively impact the mortar’s strength development.
(2) The particle sizes of graphite tailings are ten times larger than those of graphite ore, making the graphite tailings a better substitute for river sand in mortar and concrete. Notably, the optimal mechanical properties of the cementitious mortar were achieved when graphite tailings were applied as a fine aggregate into the cementitious mortar at a mixing ratio of 40%, resulting in a 28-day compressive strength of 48.8 MPa and flexural strength of 11.7 MPa.
(3) The incorporation of graphite ore will substantially improve the rheological properties of cementitious mortar. The reason for the weakening of the mechanical strength of GOM is that graphite is distributed among the gaps formed by large aggregate particles. However, the mechanism of mechanical strength enhancement of GTTM is that the voids between large-particle aggregates are filled by graphite tailings and ettringite, which adhere closely to Ca(OH)_2_ and C-A-S-H gel, fully filling the pores and making the cementing material denser and more conducive to the subsequent development of mechanical strength.

## Figures and Tables

**Table 1:** Raw materials and origin.

Raw Material	Place of Origin
Graphite ore	A field in Jixi in Heilongjiang
Graphite tailings	A field in Jixi in Heilongjiang
Fly ash	Shandong Weiqiao Group
Blast-furnace slag powder	Hebei Tangshan Iron and Steel Plant
Ordinary Portland cement	Hebei Tangshan Fushun Cement Factory
Iron tailings	A field in Hebei LuanPing
Desulfurized gypsum	Shandong Weiqiao Group

**Table 2:** Mixing proportions for producing mortars with graphite ore as a supplementary cementitious material.

Group No.	Graphite Ore/wt%	S95 Slag Powder/wt%	Fly Ash/wt%	52.5 Portland Cement/wt%	Desulfurized Gypsum/wt%	Iron Tailings/wt%
A1	0	15	10	23	2	50
A2	2	15	8	23	2	50
A3	4	15	6	23	2	50
A4	6	15	4	23	2	50
A5	8	15	2	23	2	50
A6	10	15	0	23	2	50
A7	12	13	0	23	2	50
A8	16	9	0	23	2	50
A9	20	5	0	23	2	50
A10	25	0	0	23	2	50

**Table 3:** Mixing proportions for producing mortars with graphite tailings as a supplementary cementitious material.

Group No.	Graphite Tailings/wt%	S95 Slag Powder/wt%	Fly Ash/wt%	42.5 Portland Cement/wt%	Desulfurized Gypsum/wt%	Iron Tailings/wt%
B1	0	15	10	23	2	50
B2	2	15	8	23	2	50
B3	4	15	6	23	2	50
B4	6	15	4	23	2	50
B5	8	15	2	23	2	50
B6	10	15	0	23	2	50
B7	12	13	0	23	2	50
B8	16	9	0	23	2	50
B9	20	5	0	23	2	50
B10	25	0	0	23	2	50

**Table 4:** Mixing proportions for producing mortars with graphite tailings as fine aggregates.

Group No.	Graphite Tailings/wt%	Iron Tailings/wt%	42.5 Portland Cement/wt%
C1	25	25	50
C2	30	20	50
C3	40	10	50
C4	50	0	50

**Table 5:** Basic physical properties of river sand and graphite tailings [24].

Sample	Bulk Density (kg/m^3^)	Apparent Density (kg/m^3^)	Fineness Modulus	Crush Value (%)	Porosity (%)	Water Absorption Rate (%)
River sand	1572	2588	2.2	15.04	39.38	0.48
Graphite tailings	1355	2880	1.3	27.64	45.22	1.54

## Data Availability

Data will be made available on request.
